# Evaluation of human growth hormone (somatropin) in socket healing: a split-mouth randomized controlled trial

**DOI:** 10.1097/MS9.0000000000000422

**Published:** 2023-04-03

**Authors:** Nadim Sleman, Ali Khalil

**Affiliations:** a Oral and Maxillofacial Surgery Department, Tishreen University Hospital; b Oral and Maxillofacial Surgery Department, Faculty of Dentistry, Tishreen University, Latakia, Syrian Arab Republic

**Keywords:** alveolar bone loss, alveolar bone preservation, CBCT, clinical trial, Growth hormone (somatropin), socket healing

## Abstract

Techniques for preserving alveolar bone after tooth extraction are becoming a part of the usual clinical practice of clinicians. These techniques aim at minimizing postextraction bony resorption, hence, minimizing subsequent follow-up for implant insertion. This randomized clinical study aimed to measure and compare alveolar bone and soft tissue healing between extraction sockets treated with somatropin to untreated sockets.

**Methods:**

The study is designed as a split-mouth randomized clinical trial. The selected patients were indicated for bilateral symmetrical tooth extraction, where each patient had an indication to extract two symmetrical teeth in anatomy and number of roots. Somatropin was applied to the tooth socket of the randomly selected side after tooth extraction by gel foam, and the control side was filled with gel foam only. A clinical follow-up of the soft tissue was done 7 days after tooth extraction to evaluate clinical aspects of the healing process. Radiographic follow-up was performed using a cone-beam computed tomography scan to assess volumetric changes of alveolar bone in the extraction area prior to and 3 months after the surgical procedure.

**Results:**

A total of 23 patients (aged 29.1±9.5 years) participated. The results showed a statistically significant association between somatropin application and better preservation of the bony dimensions of the alveolar ridge. Bone loss was −0.691±0.628 mm for the buccal plate on the study side compared to −2.008±1.175 mm on the control side. The level of the lingual/palatal plate bone loss was −1.052±0.855 mm on the study side compared to −2.695±1.878 mm on the control side. The bone loss of alveolar width was −1.626±1.061 mm on the study side compared to −3.247±1.543 mm on the control side. The results also showed better healing of covering soft tissues (*P*<0.05), as well as bone density in the socket where somatropin was applied, which has been statistically significant.

**Conclusion:**

The data from this study demonstrated that the application of somatropin in tooth sockets postextraction showed an effective contribution to reducing alveolar bone resorption and improving bone density following extraction, in addition to better healing of covering soft tissue.

## Introduction

Highlights

- Somatropin can make a huge difference in the concept of preimplant insertion preparation.
- Soft tissue healing improves when somatropin is added to the dental socket after extraction.
- Bone graft is not mandatory before implant insertion when the extraction is achieved with no trauma to the alveolar bone.

In recent years, the demand for replacing missing teeth with dental implants has increased, as it provides a more effective treatment option in oral rehabilitation. Bone resorption subsequent to the tooth extraction process constitutes a challenge to dental implants, as the dimensions of the alveolar edge suffer a significant decrease during the healing process after tooth extraction. This decrease in dimensions leads to a limitation in the width and height of the remaining socket. Dimensional changes that occur in the alveolar bone after dental extraction has been the scope of many studies on humans. These changes were studied according to different methodologies, including clinical study, the study of gypsum examples, and radiographic studies^1^–^6^.

Research has shown that the amount of bone absorption vertically is 11–22%, and the amount of horizontal absorption is 26.7–62.4% within 2–7 months after dental extraction^7^–^12^.

The techniques currently used to preserve the dimensions of the alveolar ridge after dental extraction depends on the principle of guided bone regeneration, which includes creating a bone space, such as the use of a barrier membrane to prevent the migration of epidermal cells to this space and thus allowing bone cells to grow within the blood clot to form new bone. Bone grafts are used to preserve the dimensions of the alveolar margin as well. They are, however, less useful without the use of membranes. Therefore, it remains better to use membranes with bone graft to preserve the alveolar edge rather than using either membranes or bone graft alone^7^.

Bone grafting techniques are usually resorted to in cases where the alveolar process suffers severe absorption in order to receive dental implants. In such cases, an autograft is used in addition to xenograft, allograft, and alloplast^8^–^16^.

The grafting process is associated with a set of disadvantages, including the pathogenesis related to the donor site (pain, edema, etc.), the length of the surgical procedure, the increased risk of infection, and the cost of the surgical procedure. In addition, it is difficult to accurately predict the size of the bone gain after the end of the healing period and the insertion of dental implants.

Therefore, it has been necessary to rely on an appropriate and predictable method to maintain the dimensions of the alveolar bone after tooth extraction.

The medicinal description of growth hormone proved effective in accelerating the process of bone regeneration and activating osteoblasts in adults^17^–^21^.

Treatment with growth hormone in adults leads to a significant increase in muscle and bone density. Patients treated with growth hormone have shown better muscle performance and improvements in their quality of life^22^.

Treatment with growth hormone in experimental animals led to an increase in cortical bone mass^23^ and an improvement in the mechanical properties of bone^20^,^21^,^23^. In addition, studies have proven growth hormone’s ability to improve the speed and quality of bone regeneration in bone defects due to direct or indirect stimulation of osteoblasts^24^,^25^.

Impact of topical growth hormone application:

A significant improvement in bone healing and maximum stability of titanium implants was observed in experimental animals due to the topical use of growth hormone^26^,^27^.

A study on rabbits demonstrated that topical application of growth hormone supported the growth of temporomandibular joint cartilage in a very short time^28^.

Bone regeneration was evaluated when topical somatropin had been applicated in repairing defects surgically created in the tibias of rabbits. The results showed that this approach supports the bone regeneration process^29^.

The purpose of the study was to evaluate the efficacy of applying topical human growth hormone in preserving the alveolar ridge after tooth extraction. The evaluation results were obtained using clinical and radiographic parameters in comparison with natural healing.

The investigators hypothesize that the human growth hormone material could be effective in preventing tissue collapse and preserving the dimensions of the alveolar ridges.

The study aims specifically at the following:

1. Evaluating the local application of human growth hormone (somatropin) in maintaining the dimensions of the socket after dental extraction.
2. Evaluating the density of the bone formed at the extraction site after the application of somatropin.
3. Evaluating soft tissue healing at the extraction site after the application of somatropin.

## Materials and methods

### Study design and sample

**Figure 1:**
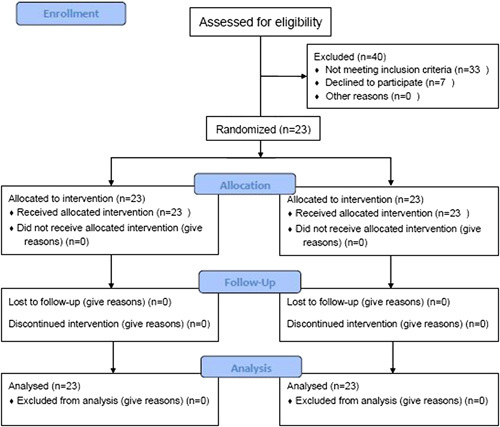
CONSORT Flowchart.

To address the research purpose, the investigators designed and implemented a split-mouth and randomized double-blind clinical trial in accordance with the Declaration of Helsinki and with the Consolidated Standards of Reporting Trials (CONSORT) statement writing guidelines for clinical trials (Fig. 1)^30^.

This study is registered with the Research Registry by the identifying number: researchregistry8677 and the reference hyperlink is https://www.researchregistry.com/browse-the-registry#home/

### Participants

The study population was composed of all patients presenting for evaluation and management of indicated bilateral tooth extraction at the University’s Hospital (Latakia, Syria) between August 2021 and May 2022. All included patients in the study were followed up for 3 months.

To be included in the study sample, patients with symmetrical tooth extraction indication (vertical root fractures – root caries – poor prognosis for endodontic treatment) of both genders over 18 years old had to be nonsmokers, clinically healthy with no systemic diseases (no diabetic patients were included). They also needed to be with an oral hygiene index of more than 80%^31^.

The participation of those patients included in the study was approved by informed consent.

Patients excluded from the study trial were pregnant women, patients who had advanced periodontitis at the extraction site, patients with chronic apical pathology, smokers, patients who suffered the loss of one or more socket walls after extraction, patients undergoing orthodontic treatment, patients with endocrine diseases that affect bone metabolism. As well as patients receiving bisphosphonates, corticosteroids, and oncology patients undergoing chemotherapy and/or radiotherapy.

### Variables

The primary predictor variable was mainly the treatment protocol in the study corresponding to either filling the socket with gel foam soaked in somatropin (study side) or gel foam without adding any substance (control side).

Primary outcome variables include:

1. Alveolar dimensions were assessed 3 months after the procedure, including bone height for the buccal plate and the palatal/lingual plate with respect to adequate reference points. Bone width has been measured from the buccal to the palatal/lingual plate at the top of the bony ridge.
2. Bone density was measured by Hounsfield units and it has been assessed 3 months after the procedure.

Secondary outcome variables include:

1. Soft tissue healing was assessed clinically 7 days after teeth extraction.

Covariates:

1. Demographic covariates included age and sex.
2. Perioperative covariates included extraction site in the mandible or maxilla, periapical lesion, and the number of roots.

### Statistical analysis

All statistical analysis was done using IBM SPSS 2020 version 27.0 software for Windows.

Paired samples *t* test was used for analyzing results to show differences between the study side and the control side. Pearson correlation was used for analyzing covariates. A *P* value less than 0.05 was typically considered to be statistically significant.

### Study sample

To calculate the required number of participants to run our study trial, we considered the data of previous review studies on growth factors in preserving alveolar ridge after tooth extraction.

Sixty-three patients presented to the University’s Hospital between August 2021 and May 2022 asking for teeth extraction. Twenty-three patients were accepted for the study.

The calculated number of participants considering a 5% error probability and 80% statistical power was 23 patients (46 sides).

**Figure 2:**
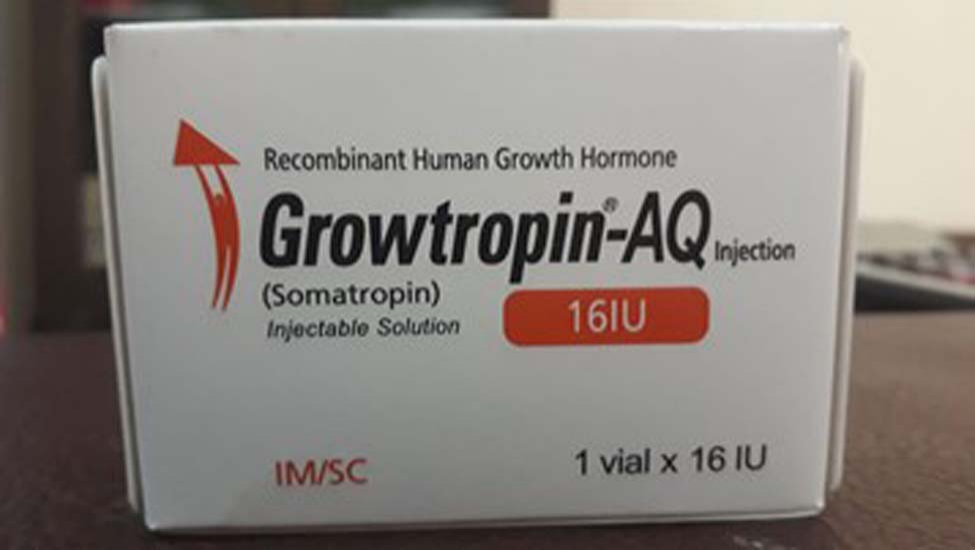
Somatropin solution.

One of the two sides of the extraction was chosen to fill the socket with the gel foam absorbing liquid somatropin 2 IU (Fig. 2). In order to decide the side of the study, the patients had to randomly select a card from a set of cards that either had number 1 or 2. Number 1 indicated the right side and number 2 the left one. This randomization was done by a single investigator. Neither the participants nor the researcher knew which side would be chosen for the somatropin application.

### Surgical procedure

**Figure 3:**
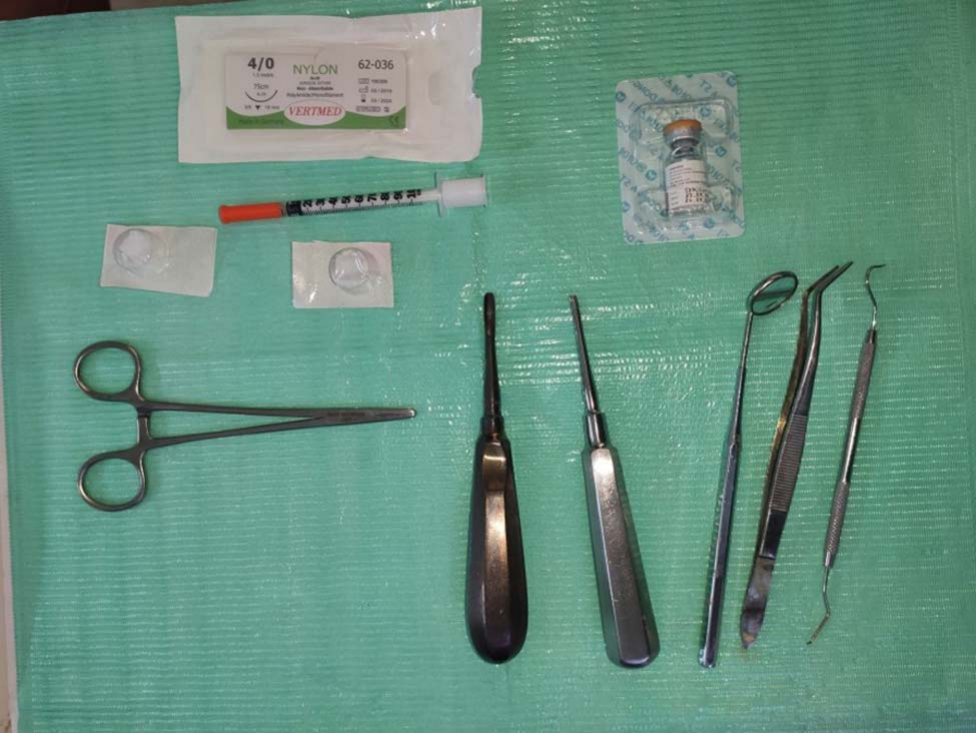
Surgical tools.

Patients rinsed out their mouths with a 0.12% chlorhexidine digluconate mouthwash (Biofresh-Mouthwash K – Mouthrinse). Lips and skin were cleaned with Povidone-iodine (Fig. 3).

Vestibular and palatal local infiltrative anesthesia using lidocaine 2% with 1:1 000 000 epinephrine (Adrecaine) was performed on the maxilla and bilateral inferior alveolar nerve block was performed on the mandible^32^.

Extraction was performed using extraction forceps and/or elevators (Synamed USA). Then a sufficient curettage of the lesion was done after extraction (Surtex).

A bilateral symmetrical extraction was performed without causing trauma to the cortical plates and without raising a mucoperiosteal flap. Hence, it was possible to apply gel foam (Gelfoam-Pfizer) to fill the sockets on both sides. Not to mention that the study side was filled with absorbing gel foam of liquid somatropin 2 IU (Growtropin-AQ). Afterward, the socket was sutured on both sides with an 8-suture (4/0 nylon SurgiReal) to fix the gel foam into the socket. No medication was required.

The sutures were removed after 7 days and a clinical assessment was done.

### Radiographic evaluation

Radiographic comparison was done using cone-beam computed tomography (CBCT) (CS9600 CBCT machine – Carestream software for Dicom viewing) to evaluate dimensions of the alveolar bone prior to the procedure and 3 months after extraction, as well as measuring bone density 3 months after the surgical procedure.

CBCT analysis:

The following dimensions were evaluated:

1. the height of the buccal plate;
2. the height of the lingual/palatal plate;
3. ridge width.

**Figure 4:**
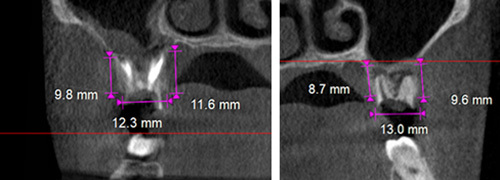
Shows the measurements of alveolar bone dimensions using CBCT scan before the surgical procedure (somatropin was applied in the socket of upper left molar).

**Figure 5:**
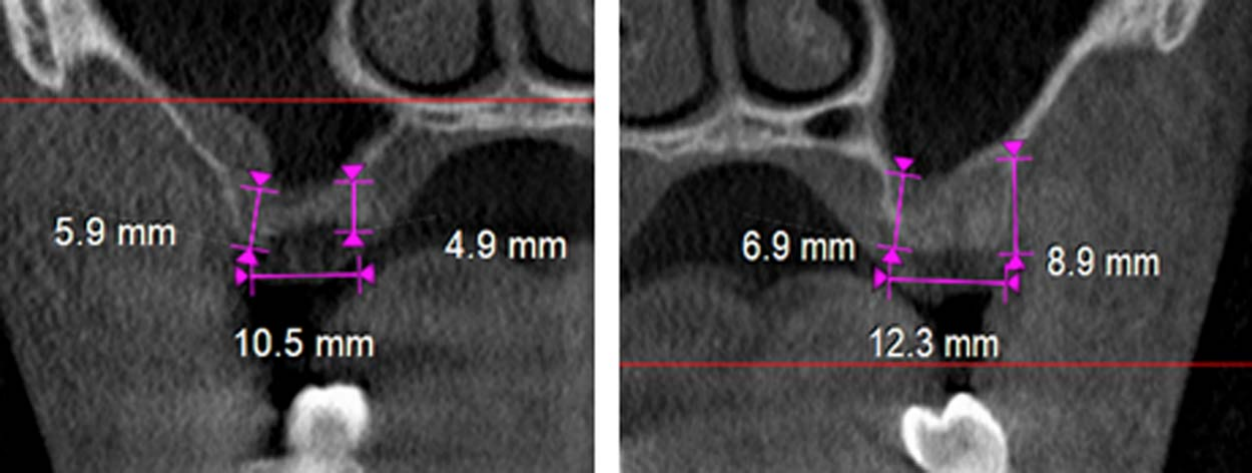
Shows the measurements of alveolar bone dimensions using CBCT scan after the surgical procedure (somatropin was applied in the socket of upper left molar.

For deciding the height of the buccal and lingual/palatal plates, measurements have been taken from the top of the buccal and lingual/palatal plate in the middle of the alveolus to the appropriate reference point (Figs. 4, 5).

Reference points for the lower jaw: the roof of the dental alveolar canal in the posterior teeth and the lower edge of the mandible in the anterior teeth.

Reference points for the upper jaw: the bottom of the nasal cavity and the bottom of the maxillary sinus.

For deciding the alveolar width, measurements have been taken between the buccal and the palatal/lingual plates at the top of the bony ridge.

Previous dimensions were measured before teeth extraction and 3 months after teeth extraction.

### Bone density analysis

**Figure 6:**
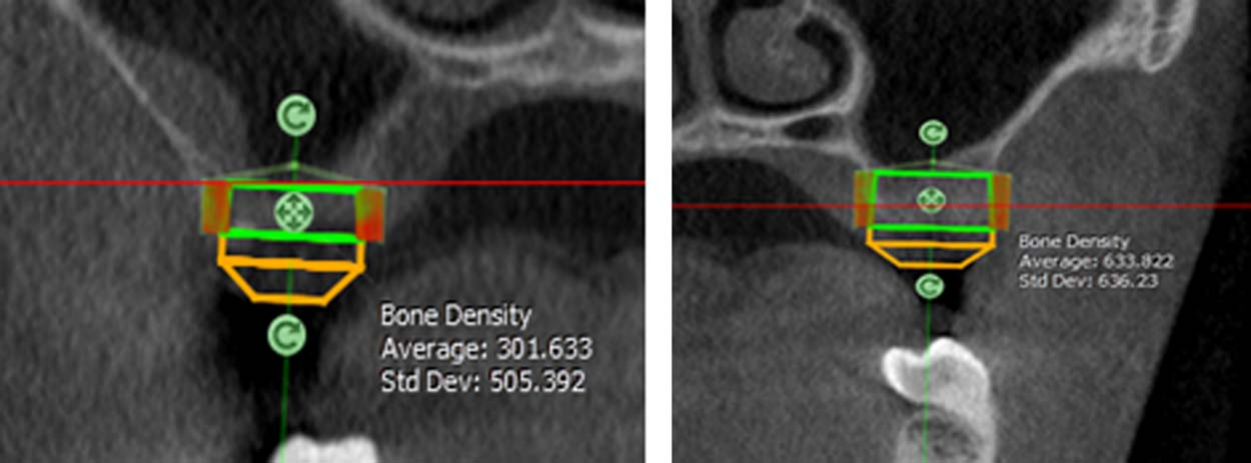
Shows measurement of bone density using CBCT scan (somatropin was applied in the socket of upper left molar.

In order to evaluate the bone density of the extracted tooth socket, a sagittal cross-section of the middle of the extracted tooth socket and the mean of all bone density points were taken after 3 months of extraction, and then the Hounsfield bone density units were calculated (Fig. 6).

### Soft tissue healing analysis

The healing index we use was proposed by Landry, Turnbull, and Howley^33^ (Table 1).

**Table 1:** Soft tissue healing index.

Parameters	Score 0	Score 1	Test site score	Control site score
Gingival color	Totally/partially red	Pink		
Granulation tissue	Present	Absent		
Epithelialization degree	Partial	Complete		
Swelling	Present	Absent		
Bleeding	Present	Absent		
Pain	Present	Absent		
Suppuration	Present	Absent		
Total score				

The following evaluation parameters were proposed for postextraction sites by applying a dichotomic score (0/1) with a total score of 7: presence/absence of redness; presence/absence of granulation tissue; presence/absence of suppuration; presence/absence of swelling; degree of tissue epithelialization (partial/complete); presence/absence of bleeding; presence/absence of pain on palpation.

Clinical assessment was performed 7 days after the procedure for the soft tissue healing and the final assessment was performed 3 months after the surgery.

## Results

Twenty-three participants in total fulfilled the inclusion criteria aged (aged 29.1±9.5 years).

No patient experienced any follow-up complications. There have been no side effects in the region where topical somatropin has been applicated.

### (1) Alveolar bone height

**Figure 7:**
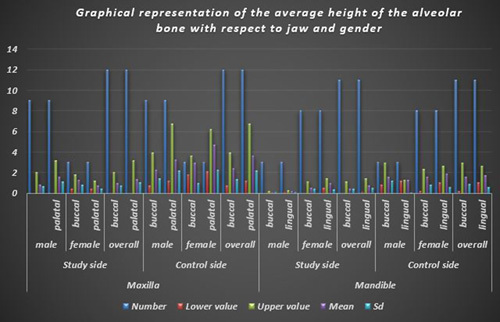
Graphical representation of the average height of the alveolar bone with respect to jaw and gender.

Table 2 shows the statistics related to the average height of the buccal and palatal/lingual plates and their upper and lower values with respect to the jaw, gender, and side. Where the bone was mostly preserved on the study side (Fig. 7).

**Table 2:** Some descriptive statistics related to the height of the alveolar bone

Jaw	Side	Gender	Plate	Number	Lower value	Upper value	Mean	SD
Maxilla	Study side	Male	Buccal	9	0	2	0.822	0.689
			Palatal	9	0	3.2	1.533	1.120
		Female	Buccal	3	0.4	1.8	1.300	0.781
			Palatal	3	0.4	1.2	0.700	0.436
		Overall	Buccal	12	0	2	0.942	0.709
			Palatal	12	0	3.2	1.325	1.044
	Control side	Male	Buccal	9	0.7	3.9	2.222	1.420
			Palatal	9	1.2	6.7	3.244	2.194
		Female	Buccal	3	1.8	3.6	2.933	0.987
			Palatal	3	2.1	6.2	4.700	2.261
		Overall	Buccal	12	0.7	3.9	2.400	1.322
			Palatal	12	1.2	6.7	3.608	2.206
Mandible	Study side	Male	Buccal	3	0	0.2	0.100	0.100
			Lingual	3	0.1	0.3	0.200	0.100
		Female	Buccal	8	0	1.1	0.538	0.403
			Lingual	8	0.5	1.4	0.963	0.370
		Overall	Buccal	11	0	1.1	0.418	0.397
			Lingual	11	0.1	1.4	0.755	0.474
	Control side	Male	Buccal	3	0.8	2.9	1.533	1.185
			Lingual	3	1.2	1.3	1.267	0.058
		Female	Buccal	8	0.2	2.3	1.600	0.804
			Lingual	8	1	2.6	1.863	0.593
		Overall	Buccal	11	0.2	2.9	1.582	0.857
			Lingual	11	1	2.6	1.700	0.569

### (2) Bone width

**Figure 8:**
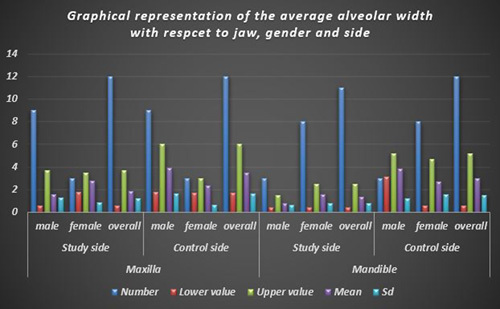
Graphical representation of the average alveolar width with respect to jaw, gender and side.

Table 3 shows the statistics related to the means, standard deviations, and the upper and lower values of the alveolar width with respect to the jaw, gender, and side. Where it turns out that bone width was preserved better on the study side (Fig. 8).

**Table 3:** Some descriptive statistics related to alveolar bone width

Jaw	Side	Gender	Number	Lower value	Upper value	Mean	SD
Maxilla	Study side	Male	9	0.60	3.70	1.589	1.240
		Female	3	1.80	3.50	2.733	0.862
		Overall	12	0.60	3.70	1.875	1.234
	Control side	Male	9	1.80	6.00	3.878	1.660
		Female	3	1.70	3.00	2.333	0.651
		Overall	12	1.70	6.00	3.492	1.603
Mandible	Study side	male	3	0.40	1.50	0.800	0.608
		Female	8	0.40	2.50	1.563	0.802
		Overall	11	0.40	2.50	1.355	0.807
	Control side	Male	3	3.10	5.20	3.833	1.185
		Female	8	0.60	4.70	2.663	1.553
		Overall	12	0.60	5.20	2.982	1.506

### (3) Bone density

**Figure 9:**
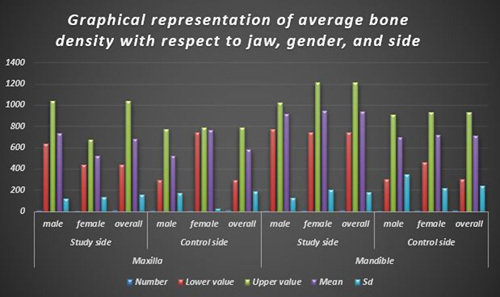
Graphical representation of average bone density with respect to jaw, gender, and side.

Table 4 shows statistical values of bone density with respect to the jaw, gender, and side, as it turns out that the bone density was higher on the study side (Fig. 9).

**Table 4:** Some descriptive statistics related to alveolar bone density

Jaw	Side	Gender	Number	Lower value	Upper value	Mean	SD
Maxilla	Study side	Male	9	0.60	3.70	1.589	1.240
		Female	3	1.80	3.50	2.733	0.862
		Overall	12	0.60	3.70	1.875	1.234
	Control side	Male	9	1.80	6.00	3.878	1.660
		Female	3	1.70	3.00	2.333	0.651
		Overall	12	1.70	6.00	3.492	1.603
Mandible	Study side	Male	3	0.40	1.50	0.800	0.608
		Female	8	0.40	2.50	1.563	0.802
		Overall	11	0.40	2.50	1.355	0.807
	Control side	Male	3	3.10	5.20	3.833	1.185
		Female	8	0.60	4.70	2.663	1.553
		Overall	12	0.60	5.20	2.982	1.506

### (4) Soft tissue healing

Table 5 shows statistical values of healing scores a week after treatment with respect to the jaw, gender, and side, as it turns out that soft tissue healing was better on the study side.

**Table 5:** Healing scores a week after the treatment

Jaw	Maxilla						Mandible					
Side	Study side			Control side			Study side			Control side		
Gender	Male	Female	Overall	Male	Female	Overall	Male	Female	Overall	Male	Female	Overall
Gingival color												
Present	7	0	7	8	2	10	2	4	6	3	8	11
Absent	2	3	5	1	1	2	1	4	5	0	0	0
Granulation tissue												
Present	0	1	1	9	3	12	1	1	2	3	8	11
Absent	9	2	11	0	0	0	2	7	9	0	0	0
Epithelialization degree												
Partial	0	1	1	9	3	12	2	0	2	3	8	11
Complete	9	2	11	0	0	0	1	8	9	0	0	0
Swelling												
Present	0	0	0	5	1	6	0	0	0	2	2	4
Absent	9	3	12	4	2	6	3	8	11	1	6	7
Bleeding												
Present	0	0	0	3	0	3	0	0	0	3	8	11
Absent	9	3	12	6	3	9	3	8	11	0	0	0
Pain												
Present	0	0	0	1	0	1	0	0	0	1	2	3
Absent	9	3	12	8	3	11	3	8	11	2	6	8
Suppuration												
Present	0	0	0	0	0	0	0	0	0	0	0	0
Absent	9	3	12	11	1	12	3	8	11	3	8	11

There has been a great speed in the healing of the soft tissues in the area of application of the material compared to the control side when removing the surgical sutures (after 7 days).

**Figure 10:**
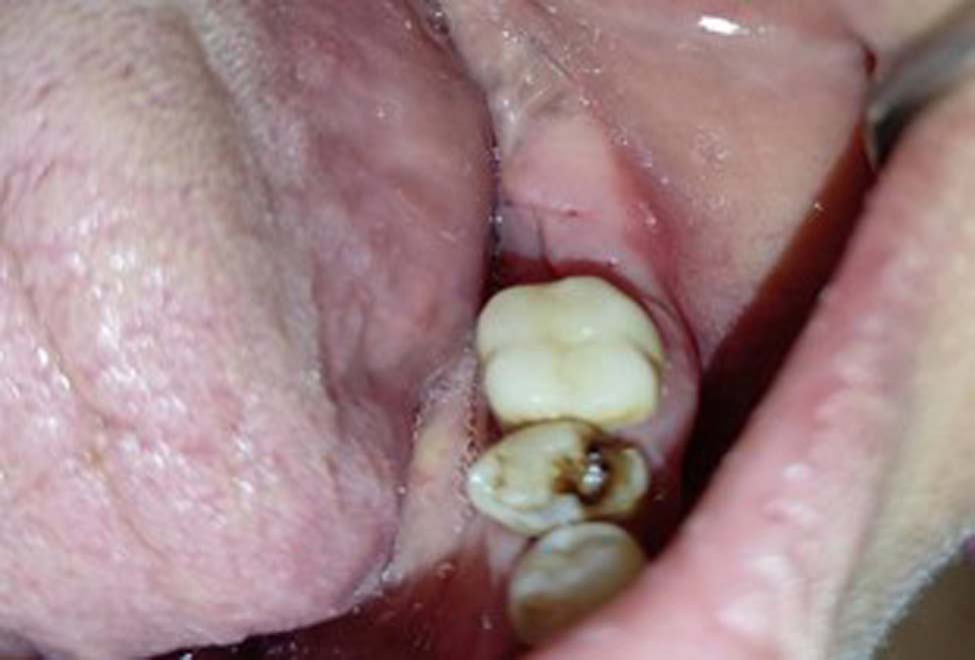
Soft tissue healing 7 days after the surgical procedure ( no flaps were raised for closure).

There have been no signs of bleeding, pain, swelling, and suppuration in the area of somatropin application, with a preference for tissue restoration better than that of the control side (Fig. 10).

Tissue healing was noticed 7 days after the surgical procedure (no flaps were raised for closure).

### Data analysis

According to the comparison between the study side and the control side (Table 6), it is clear that there have been significant *P* values less than 0.05, differences in the average loss in the buccal, lingual/palatal plates, and alveolar width between the two groups. As well as there have been significant differences, *P* values greater than 0.05, in the average bone density between the two groups.

**Table 6:** Comparison of study variables with respect to the predictor variable (treatment)

	Paired differences							
				95% CI of the difference				
Alveolar bone dimensions	Mean	SD	SEM	Lower	Upper	*t*	df	Sig. (2-tailed)
Height change of buccal plate	1.3174	1.265	0.264	0.770	1.864	4.995	22	0.000
Height change of palatal/lingual plate	1.6435	1.8456	0.385	0.845	2.442	4.271	22	0.000
Width change	1.6217	1.76325	0.368	0.859	2.384	4.411	22	0.000
Bone density	−162.316	221.121	46.107	−257.936	−66.70	−3.520	22	0.002
Soft tissue healing	−3.3044	0.97397	0.203	−3.72552	−2.883	−16.271	22	0.000

The results of soft tissue healing have shown superiority in the study group compared to the control group, indicating that the application of somatropin has had the advantage of reducing inflammation signs.

Table 7 shows the summary of covariates with respect to treatment outcomes:

1. A negligible correlation between treatment groups and sex has been detected in buccal plate height (0.004), palatal/lingual plate height (0.064), bone width (−0.131), bone density (0.242), and soft tissue healing (0.058).
2. A negligible correlation between treatment groups and age has been detected in buccal plate height (0.020), palatal/lingual plate height (−0.090), bone width (−0.168), and soft tissue healing (−0.150). In contrast, a low positive correlation has been noted in bone density (0.436).
3. A negligible correlation between treatment groups and jaw type has been detected in buccal plate height (−0.296), alveolar bone width (−0.168), and soft tissue healing (−0.150). In contrast, a low negative correlation has been noted in palatal/lingual plate (−0.376) and a low positive correlation in bone density (0.436).
4. A moderate negative correlation between treatment groups and the periapical lesion has been noted in buccal plate height (−0.632) and palatal/lingual plate height (−0.611). In contrast, a low positive correlation has been detected in bone density (0.328), and a negligible correlation has been noted in bone width (0.071) and soft tissue healing (0.119).
5. A negligible correlation between treatment groups and the number of roots has been noted in buccal plate height (0.245), bone width (0.047), bone density (−0.161), and soft tissue healing (0.112). In contrast, a low positive correlation has been noted in palatal/lingual plate height (0.348).

**Table 7:** Summary of covariates with respect to treatment outcomes

	Buccal plate height	Palatal/lingual plate height	Alveolar bone width	Bone density	Soft tissue healing
Gender					
Pearson’s correlation	0.004	−0.064	−0.131	0.2420	0.0580
Sig. (2-tailed)	0.980	0.6720	0.3840	0.1050	0.7010
*N*	46	46	46	46	46
Age					
Pearson’s correlation	0.020	−0.090	0.165	−0.041	0.103
Sig. (2-tailed)	0.898	0.552	0.274	0.786	0.494
*N*	46	46	46	46	46
Jaw					
Pearson’s correlation	−0.296^*^	−0.376^*^	−0.168	0.436^**^	−0.150
Sig. (2-tailed)	0.046	0.0100	0.263	0.002	0.321
*N*	46	46	46	46	46
Periapical lesion					
Pearson’s correlation	−0.632^**^	−0.611^**^	0.071	0.328^*^	0.119
Sig. (2-tailed)	0.000	0.000	0.637	0.026	0.432
*N*	46	46	46	46	46
Number of roots					
Pearson’s correlation	0.245	0.348^*^	0.047	−0.161	0.112
Sig. (2-tailed)	0.101	0.018	0.758	0.286	0.459
*N*	46	46	46	46	46

*Correlation is significant at the 0.05 level.

**Correlation is significant at the 0.01 level.

## Discussion

Alveolar bone preservation is a necessary procedure to reduce bone and soft tissue loss after tooth extraction. It could be performed immediately after tooth extraction. Studies have found that procedures to preserve the alveolar process after tooth extraction results in greater bone-tissue dimensions in the mouth and face compared to cases where the alveolar bone was not preserved from subsequent resorption^34^.

Our study agrees with the literature review of Pranskunas *et al.* 35 on the use of growth factors and mesenchymal cells in order to preserve alveolar bone from subsequent resorption after tooth extraction.

Our study agrees with the study of Zanettini *et al*.^36^ on reducing bone resorption in the dimensions of the alveolar bone 3 months after tooth extraction, in which he applied growth hormone by gel foam in the extracted tooth alveoli on the upper jaw of a 39-year-old female patient.

Our study also agrees with the study of Giacomin *et al*.^37^ in preserving the dimensions of the alveolar bone in which he applied Bio-Oss with growth hormone to a patient who had a second premolar indicated for extraction on the upper jaw. Four months later, the results showed a preference for applying the material in preserving the dimensions of the alveolar bone.

Castro *et al*.’s study^38^ had an advantage over our study in preserving the buccal plate after tooth extraction in the anterior region of the upper jaw. In Castro *et al*.’s study, the socket was filled with a leukocyte and platelet-rich fibrin (L-PRF), and the absorption was −0.2 mm 3 months after tooth extraction, while the bone resorption at the level of the buccal plate in this study was −0.691 mm, and the difference can be explained as follows:

1. Strauss *et al*.^39^ suggested that the L-PRF template plays an important role in suppressing the activity of osteoclasts.
2. Our study dealt with the multi-rooted teeth in both jaws, while Castro *et al*.’s study dealt only with the single-rooted teeth in the anterior region of the upper jaw. Keeping in mind that the alveolar bone suffers a relatively greater absorption with the extraction of multi-rooted teeth than it does with the extraction of single-rooted teeth.

However, our study agrees with Castro *et al*.’s study^38^ in the preservation of the palatal plate dimensions after tooth extraction in the anterior region of the upper jaw, where the absorption at the level of the palatal plate reached −1.1 mm as estimated in Castro *et al*.’s study compared with −1.052 mm in this study.

Our study agrees with Castro *et al*.^38^ in preserving alveolar width after applying L-PRF in the socket of the upper anterior teeth. When measured by CBCT after 3 months, the absorption at the level of the alveolar edge width was −2.2 mm, while in this study it was −1.626 mm. Thus, this study has an advantage in preserving the width of the alveolar ridge.

Our study agrees with the study of Cochran *et al*.^40^ in preserving the height of the alveolar process in which 0.43 mg/ml rhBMP-2 has been applied by gel foam in the extracted tooth socket. The CBCT radiographic examination 4 months later showed bone loss at the level of the height of the alveolar bone, which reached −0.8 mm, and bone loss at the level of alveolar bone width of −3.6 mm. The preference in our study can be explained by studying teeth in both jaws in the study sample, while the Cochran study was limited to upper premolars and anterior teeth.

Bone density has been higher on the side of somatropin application, at a rate of 805.198 HU, while bone density on the control side has been at an average of 642.882 HU, which means that somatropin shows an increase in bone density with a statistical significance.

Our study agrees with Martin-Monge *et al*.^41^ in his study on two groups of rabbits in which he applied growth hormone topically with a titanium implant on one of the two groups. The results have shown greater proximity of the bone to the surface of the implant when applying human growth hormone compared to the control group, while there have been no significant differences in bone density level.

Our study also agrees with Muñoz *et al*.^27^ in their study on 12 dogs in which he inserted 48 titanium implants on the lower jaw and applied 4 UI of growth hormone with 1.2 mg of melatonin before inserting the implants on the study side with no application of any substance on the control side. There were no significant differences in bone density around titanium implants.

Our study showed better healing of the covering soft tissue in extraction sites where somatropin was applicated and it agrees with a study by Zanettini *et al*.^36^ as he showed a good healing process of soft tissue after 7 days of follow-up with no signs of inflammation or infection noted.

Previous reports were a start for studying human growth hormones through case reports. This study is novel, and the clinical variations of soft tissue healing were observed 7 days after the procedure. Dimensional changes and bone density of the alveolar bone were determined using CBCT 3 months after the procedure.

The split-mouth study design gave us the advantage of making the patients their own control.

Both the patient and investigator who took the measurements were blinded to avoid researcher bias.

The limitations of this study include the difficulty of controlling the anatomy of indicated bilateral teeth to be extracted, therefore, the size and shape of studied sockets.

In addition, there were the difficulties of strictly controlling oral hygiene in all studied cases and the lack of a histological study of the newly formed bone in the extraction area 3 months after the procedure.

## Conclusions

The application of growth hormone effectively contributes to reducing alveolar bone resorption following the extraction process, as it can be said that preparing for dental implants using somatropin reduces the need for subsequent bone grafting procedures. A new concept in treatment planning should be followed as dental implants have proven to be the best option to restore lost teeth rather than all kinds of prosthodontics. The simple somatropin application after dental extraction makes it helpful to prepare for better treatment options. Furthermore, applying growth hormone topically also helps to reduce healing time until full recovery.

Paper citation^42^.

## Ethical approval

Board Name: Scientific Research Board Resolution – Tishreen University Ethics Committee, Latakia, Syria. Board Status: Approved – Approval Number: 2276; date: 23 July 2020.

## Patient consent

Written informed consent was obtained from the patients for publication of this study and accompanying images. A copy of the written consent is available for review by the Editor-in-Chief of the journal.

## Sources of funding

The study had no external sources of funding and therefore was in no way influenced by any sponsorship. Self-funded, no sponsors involved.

## Author contribution

N.F.S.: writing the manuscript, data collection, and data analysis; A.K.: supervisor.

## Conflicts of interest disclosure

The authors confirm that there were no conflicts of interest.

## Guarantor

Dr Nadim F. Sleman.

## Provenance and peer review

Not commissioned, externally peer-reviewed
